# *Dualmarker*: a flexible toolset for exploratory analysis of combinatorial dual biomarkers for clinical efficacy

**DOI:** 10.1186/s12859-021-04050-6

**Published:** 2021-03-17

**Authors:** Xiaopeng Ma, Ruiqi Huang, Xikun Wu, Pei Zhang

**Affiliations:** BeiGene (Beijing) Co., Ltd., Beijing, China

**Keywords:** Combinatory dual biomarker, Logistic regression, Cox model, R package

## Abstract

**Background:**

An increasing number of clinical trials require biomarker-driven patient stratification, especially for revolutionary immune checkpoint blockade therapy. Due to the complicated interaction between a tumor and its microenvironment, single biomarkers, such as PDL1 protein level, tumor mutational burden (TMB), single gene mutation and expression, are far from satisfactory for response prediction or patient stratification. Recently, combinatorial biomarkers were reported to be more precise and powerful for predicting therapy response and identifying potential target populations with superior survival. However, there is a lack of dedicated tools for such combinatorial biomarker analysis.

**Results:**

Here, we present *dualmarker*, an R package designed to facilitate the data exploration for dual biomarker combinations. Given two biomarkers, *dualmarker* comprehensively visualizes their association with drug response and patient survival through 14 types of plots, such as boxplots, scatterplots, ROCs, and Kaplan–Meier plots. Using logistic regression and Cox regression models, *dualmarker* evaluated the superiority of dual markers over single markers by comparing the data fitness of dual-marker versus single-marker models, which was utilized for de novo searching for new biomarker pairs. We demonstrated this straightforward workflow and comprehensive capability by using public biomarker data from one bladder cancer patient cohort (IMvigor210 study); we confirmed the previously reported biomarker pair TMB/TGF-beta signature and CXCL13 expression/ARID1A mutation for response and survival analyses, respectively. In addition, *dualmarker* de novo identified new biomarker partners, for example, in overall survival modelling, the model with combination of HMGB1 expression and ARID1A mutation had statistically better goodness-of-fit than the model with either HMGB1 or ARID1A as single marker.

**Conclusions:**

The *dualmarker* package is an open-source tool for the visualization and identification of combinatorial dual biomarkers. It streamlines the dual marker analysis flow into user-friendly functions and can be used for data exploration and hypothesis generation. Its code is freely available at GitHub at https://github.com/maxiaopeng/dualmarker under MIT license.

## Background

With the rapid development of the biomarker detection technology, an increasing number of single predictive or diagnostic biomarkers have been identified and validated in retrospective studies or perspective trials. For example, in the revolutionary field of immune checkpoint blockade (ICB) [1–3], such biomarkers include PDL1 protein level, gene expression profiling (GEP), gene mutation, and tumor mutation burden (TMB). However, cancer development is associated with a complicated tumor microenvironment (TME) and tumor/TME interactions involving multiple biological processes [1], such as tumor proliferation, apoptosis, and angiogenesis. Indeed, each single biomarker can only reflect a small aspect of these intricate tumor and immune characteristics. So far, no single perfect marker has been developed for patient stratification for ICB therapy [2].

In general, a combinatorial biomarker strategy that takes different TME-affecting components into account is needed [3]. Several combinatorial biomarkers have been identified in recent years. For example, in one pan-cancer study, the combination of TMB and the T cell–inflamed gene expression profile (TIS) was able to predict response to pembrolizumab [4]. In the Imvigor210 trial, TMB plus the TGF-beta gene signature predicted response to atezolizumab in urothelium carcinoma (UC) [5]. A third study using the same dataset found that AIRD1A mutation in combination with CXCL13 gene expression predicted overall survival (OS) [6]. In all these studies, combinatorial dual markers outperformed single markers in the prediction of response and survival.

The R language and its powerful packages for statistics and data visualization are widely used in biomarker data analysis, including the *survival* and *survminer* [7] package for survival analysis and *gClinBiomarker* [8] for univariate biomarker analysis. However, tools for dual biomarker analysis are not available to our knowledge.

Here, we describe *dualmarker*, a flexible toolset for combinatorial dual marker visualization and identification. *dualmarker* performs dual marker analysis via two distinct modules, one for in-depth evaluation of one specific biomarker pair and a second for de novo identification of biomarker pairs. Both modules are applicable for both categorical and continuous biomarkers using logistic regression and Cox survival models. We herein demonstrate its application using public biomarker data from the IMvigor210 clinical study [5] by validation of reported biomarker pairs and the identification of novel pairs.

## Implementation

### Overview

**Fig. 1:**
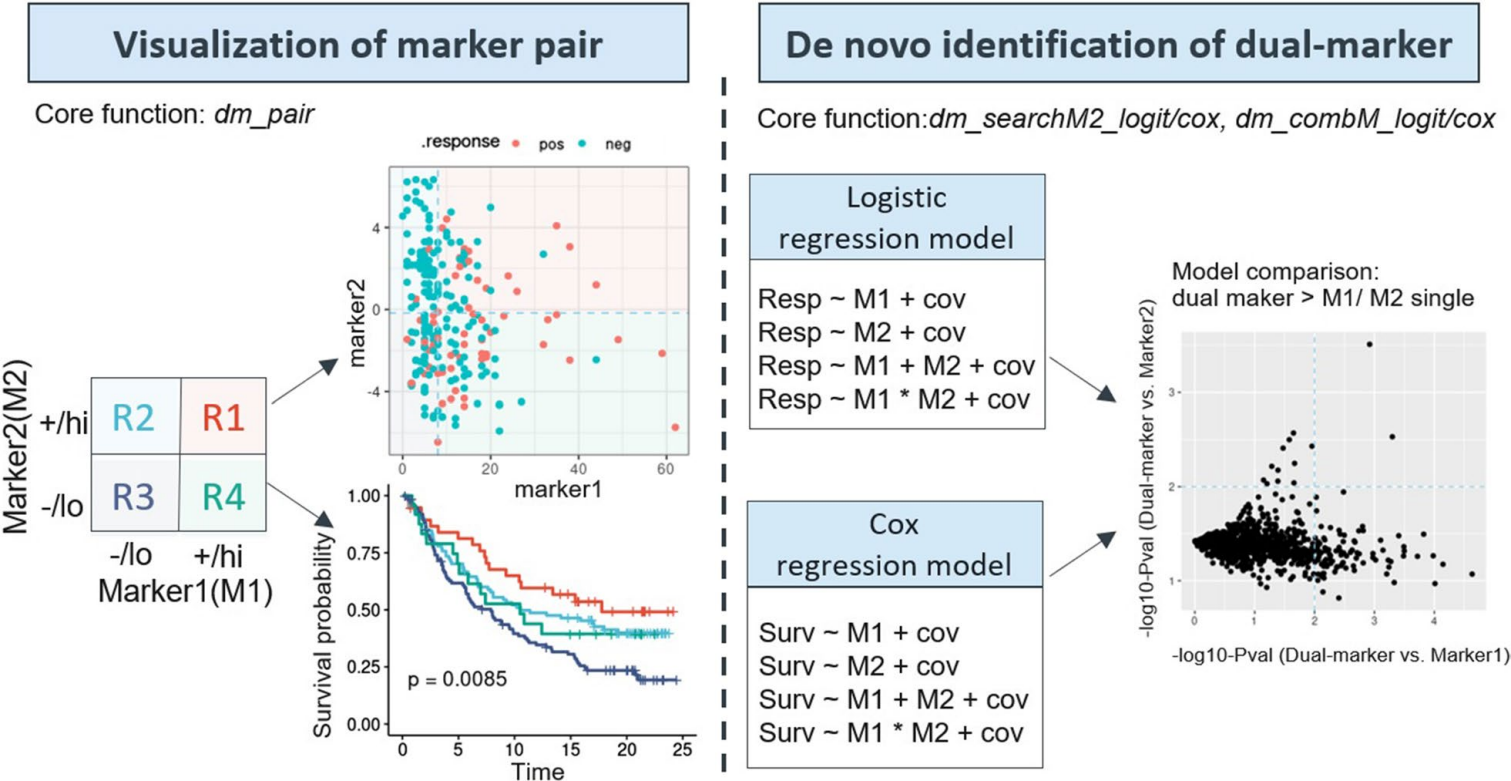
Framework of *dualmarker. Dualmarker* contains two parts: visualization of one specific marker pair and de novo identification of biomarker pairs for binary outcome (therapy response, upper left panels) and time-to-event outcome (survival, lower left panels). To facilitate visualization, both markers are dichotomized into positive/high and negative/low groups with four quadrants, labeled R1-R4 with dedicated colors. Both markers are shown in a scatter chart or Kaplan–Meier plot (KMplot, see detailed plots in Figs. 2 and 3), and all plots can be easily generated by a single function: *dm_pair*. For de novo identification of dual-marker pair, four logistic regression models or Cox regression models are built depending on whether response or survival analysis is performed: two single-marker models and two dual-marker models with interaction (M1*M2) or without interaction (M1 + M2). To control any covariates, ‘cov’ option can also be added in the model. Each candidate is evaluated by model comparison, which compares the dual-marker models with the single ones using the likelihood ratio test (LRT). The -log10 *p* values from model comparisons of dual-vs-marker1 and dual-vs-marker2 are shown as scatterplots, with each dot representing a candidate biomarker pair (see detailed plots in Fig. 4); The analysis is performed by the *dm_searhM2_logit/cox* and *dm_combM_logit/cox* functions to search marker2 to combine with specific marker1 or search among all combinations of marker pairs, respectively

*Dualmarker* assesses the correlation between biomarkers and clinical efficacy, including binary outcome (e.g. drug response) and survival data. It can evaluate a specific biomarker pair by comprehensive visualization and statistical analysis and searches for novel pairs from all dual marker combinations; It can also search for a second marker partner, namely, marker2 (M2) in combination with the chosen marker1 (M1) via logistic regression and the Cox regression model (Fig. 1). The response status should be dichotomous to fit the logistic regression model, and marker1 or marker2 can be either a continuous variable (e.g., TMB, gene expression) or a dichotomous variable (e.g., gene mutation, mutated or wild-type). Ordinal variables with more than 2 levels are not supported in the current version. Continuous variables are used directly in the logistic/Cox regression model, and they are dichotomized to high and low levels, using the population median as a cutoff or user-specified value, to facilitate visualization. The dichotomization and combination of two markers produces four subgroups, each located in a quadrant, labeled as R1-R4 with dedicated colors throughout all plots (Fig. 1).

### Visualizations

**Fig. 2:**
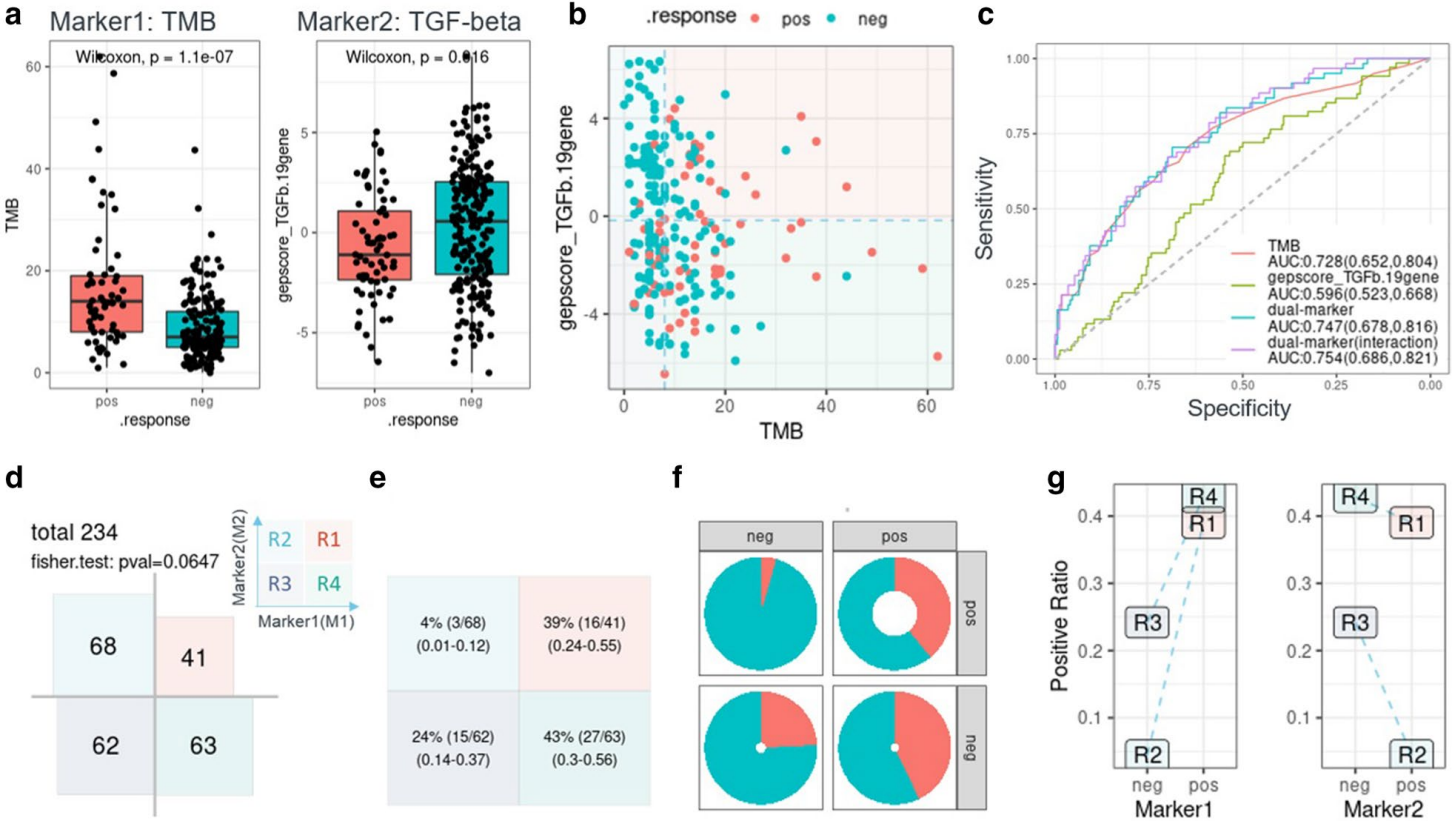
Demonstration of biomarker pairs for response analysis using TMB (marker1) and the TGF-beta signature (marker2). Boxplots display the distribution of the biomarkers (left: TMB; right: TGF-beta) by response group (positive vs negative); Wilcoxon tests were employed to compare the response group for each biomarker (**a**). The scatterplot displays the inter-marker correlation with the color representing the response category, and red indicates positive (**b**). The ROC curve depicts the performance of the single marker and dual marker using a logistic regression model with or without marker interaction. The AUC and its confidence interval are presented on the plot as well (**c**). Samples were stratified into 4 groups/quadrants, and statistics for each group are shown (**d**–**g**). The area proportion chart shows the group size and independence test of two markers by Fisher’s exact test (**d**). The quadrant statistic matrix presents the group size, response rate and confidence interval (**e**). The doughnut plot illustrates both the relative group size (width of ring) and response rate (red arc fraction, **f**). The line chart shows the response rate in each quadrant/group and the potential statistical interaction between two markers, i.e., if two lines are crossed (**g**). All figures were generated by *dualmarker* (v0.1.0)

**Fig. 3:**
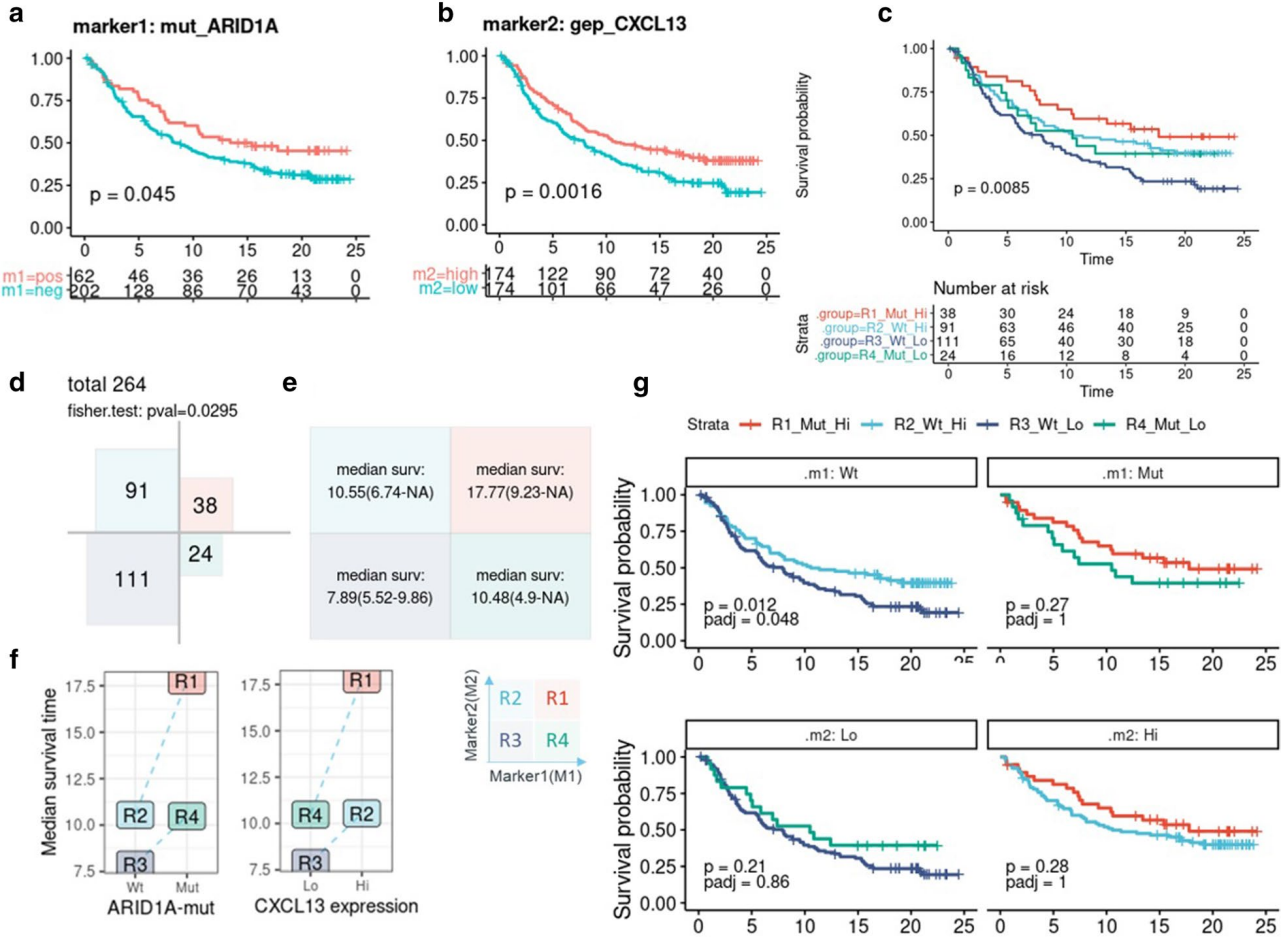
Demonstration of survival analysis for one pair of markers, ARID1A mutation (marker1) and CXCL13 gene expression (marker2). KM plot of a single marker for mut_ARID1A (**a**) and CXCL13 expression (cut by median, **b**) and the dual markers (**c**). Four group sizes and independence tests using Fisher’s exact test (**d**), the median survival time and confidence interval in each group/quadrant are shown in a matrix (**e**) and line chart (**f**). Conditional KM plots show the survival curves of marker2 stratified by the marker1 level (**g**, upper panel) as well as marker1 within the context of the marker2 level (**g**, lower 2 panel) with the *p* values of log-rank test and adjusted *p* values by ‘Bonferroni’ method. All figures were generated by *dualmarker* (v0.1.0)

*Dualmarker* provides comprehensive visualizations of dual markers and reveals their association with response and survival. For response analysis, boxplots illustrate the correlation between response and a single marker (Fig. 2a), and a scatterplot shows the inter-marker correlation (Fig. 2b). In turn, the ROC curve, AUC and confidence interval demonstrate the performance of response prediction for single markers and logistic regression model of dual markers (Fig. 2c). In addition, a series of four-quadrant plots reveals the group/quadrant size and the response rate of each group/quadrant, including an area proportion chart (Fig. 2d), a quadrant statistic matrix (Fig. 2e), a doughnut plot (Fig. 2f) and a line chart (Fig. 2g, see the legend for a detailed description). For survival analysis, a Kaplan–Meier plot (KMplot) depicts the survival of a single marker and dual markers in four groups/quadrants, with a *p* value determined by the log-rank test (Fig. 3a–c). Conditional KMplots represent the survival curve of marker1 on the condition of marker2 levels (positive/high or negative/low) and the survival curve of marker2 on the condition of marker1 levels (Fig. 3g), revealing the association between survival and marker1 in the context of marker2 levels and vice versa. In addition, similar four-quadrant plots show the group/quadrant size, median survival time and 95% confidence interval by an area proportion chart (Fig. 3d), a quadrant statistic matrix (Fig. 3e) and a line chart (Fig. 3f, the Fig. 3 legend contains detailed description of each chart). Both the response and survival visualizations are easily performed by the *dm_pair* function, which will generate over 14 plots simultaneously. In addition, there are flexible options in *dm_pair* to adjust labels, titles as well as the color schemes from palettes requested by the leading scientific journals (via R package *ggsci* [9]) or by customer specification. Taken together, this panel of charts reveals the potential association between two markers, response and survival, in an intuitive and comprehensive manner.

### Logistic regression model

The logistic regression method was applied to predict the binary outcome of response. Different modeling strategies were considered to include one biomarker at a time, two biomarkers at the same time and the modeling interaction of the two biomarkers using the following four logistic regression models: model1, Resp ~ M1; model2, Resp ~ M2; model3, Resp ~ M1 + M2; and model4, Resp ~ M1 + M2 + M1:M2 (interaction term). Covariates can also be added in the models (Fig. 1). Model comparisons were performed to evaluate the superiority of the dual-marker to single-marker model using the likelihood ratio test (LRT) with the R *anova* function. The performance of responder/non-responder classification of the biomarker(s) was evaluated using ROC/AUC by R package *pROC* [10].

### Cox survival model

The Cox regression method was applied to predict survival. Four Cox regression models were produced, similar to the logistic regression models. The parametric part of the Cox model design is as follows: Model1, Surv ~ M1; Model 2, Surv ~ M2; Model 3, Surv ~ M1 + M2; Model 4, Surv ~ M1 + M2 + M1:M2 (interaction). Covariates can also be added in the models (Fig. 1). As above, model comparisons were performed using the likelihood ratio test (LRT) implemented in R. The performance of event risk classification was evaluated using concordant probability by R package *CPE* [11].

### Search for biomarker pairs

**Fig. 4:**
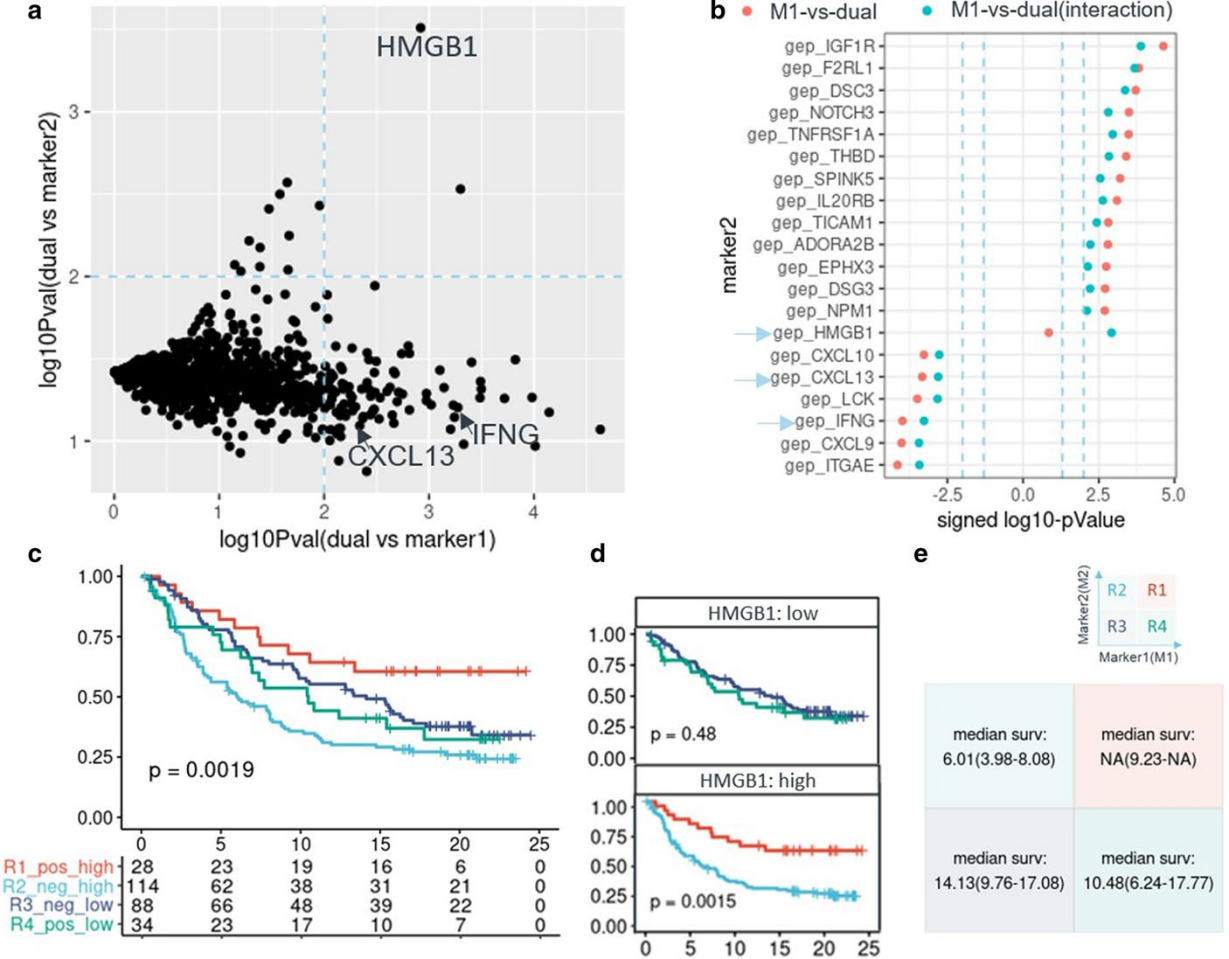
De-novo identification of marker2 from gene expression to combine with marker1 (mut_ARID1A). The dual marker Cox regression model was compared with the single marker model using marker1 and marker2 separately. The -log10 *p* values of the LRT test between models are shown on the x-axis and y-axis, and the dashed line indicates the *p* value = 0.01 (**a**). Top significant marker2s to combine with mut_ARID1A according to the significance of LRT in model comparison of the dual-marker model versus the mut_ARID1A single-marker model; dashed lines show *p* value = 0.01 and 0.05(**b**). The signed -log10-p value is provided, and the sign is the direction of the effect of marker2 on OS as a single marker. The arrow points to the known biomarker partners, CXCL13, IFNG and the novel gene HMGB1, which showed a statistical interaction with mut_ARID1A (**c-e**). Four quadrants/groups divided by mut_ARID1A and HMGB1 expression levels (high vs low, cut by population median) had significantly different OS values (**c**, *p* value = 0.0019, log-rank test for all group comparisons). ARID1A-mutated patients had longer OS under the condition of high HMGB1 but not low HMGB1 (**d**). Median survival time and confidence interval for each quadrant/group (**e**)

Based on the characterization of a single pair of biomarkers, we expanded the scope to biomarker partner searches. Using the models above, we searched for marker M2 to combine with marker M1 through the *dm_searchM2_logit* and *dm_searchM2_cox* functions, prioritizing marker2s with significant improvement in model fit in the dual-marker model versus the single-marker model. Candidate marker2s with significant LRT test *p* values are shown in a dot chart, which displays the signed -log10 *p* values, whereby the sign is the direction of the marker2 effect on response or survival as a single marker (Fig. 4b). The models can also take covariates into consideration (Fig. 1) and adjust the *p* value for multiple comparisons. Furthermore, we expanded the scope to search all biomarker pairs, using *dm_combM_logit* and *dm_combM_cox* functions. Users can filter and get interesting dual-marker pairs using plenty of statistics and model performance metrics, for example, AUC for binary response analysis (logistic regression), concordant probability estimate (CPE) for survival analysis (Cox regression), *p* value of dual-vs-single model comparison and statistical interaction of two markers, et al. In summary, *dualmarker* package can search dual-marker pairs with or without given marker.

## Results

We applied *dualmarker* to analyze the biomarker data of the IMvigor210 trial [5], including the baseline characterization of PDL1 IHC, gene expression profiling (GEP), tumor mutation burden (TMB) and gene mutations in 348 patients with advanced UC. To simplify the demonstration, for GEP, we focused on the tumor microenvironment-related genes (1392) listed in the HTG Precision immune-oncology panel [12].

### Response analysis of one biomarker pair

We explored one previously reported biomarker pair, TMB and the TGF-beta gene expression signature [5]. TMB was significantly higher in the responders than in the non-responders (Wilcoxon test, *p* value = 1.1e-7), whereas the TGF-beta gene signature score showed the opposite trend (Wilcoxon test, *p* value = 0.016, Fig. 2a). There was a weak negative correlation between TMB and the TGF-beta signature (Fig. 2b, Spearman correlation R = − 0.16, *p* value = 0.007). Using the population median as the cutoff point, patients were stratified into 4 groups/quadrants, and a comparison of the 4 group sizes showed weak dependence between TMB and the TGF-beta signature (*p* value = 0.06, Fisher exact test, Fig. 2d). The TMB-high/TGF-beta-low group (quadrant R4) had the highest response rate (43%, 95% CI 0.3–0.56); the TMB-low/TGF-beta-high group (quadrant R2) had the lowest response rate (4%, 95% CI 0.01–0.12, Fig. 2e–g). This indicated that the R2 group was most refractory to PDL1 therapy among the four groups. In addition, logistic regression models were applied to predict response, and the two biomarker combinations yielded higher AUC values than the single TMB marker (AUC = 0.754, 95% CI 0.686–0.821 for the TMB*TGF-beta model with the biomarker interaction versus 0.728, 95% CI 0.652–0.804 for the single TMB marker model, Fig. 2c). Besides, the dual marker model (with marker interaction) had statistically better goodness-of-fit than the TMB single marker model using LRT test (*p* value = 0.0098).

### Survival analysis of one biomarker pair

Dual marker survival analysis was carried out using the pair of ARID1A mutations and CXCL13 gene expression as an example. This biomarker pair was reported to be associated with OS [6]. We confirmed that patients with ARID1A mutations (marker1) had a longer OS (*p* value = 0.045, log-rank test, Fig. 3a; patients with higher expression of CXCL13 (marker2, population median as cutoff) also had a longer OS (*p* value = 0.0016, log-rank test, Fig. 3b). We then stratified the population into four groups/quadrants and observed a significant difference in survival (*p* value = 0.0085, log-rank test for comparison of all groups, Fig. 3c), with the R1 group (ARID1A-mut/CXCL13-high) showing the longest OS (median 17.7, CI 9.23-NA) and the R3 group (ARID1A-wt/CXCL13-low) the shortest OS (median 7.89, CI 5.52–9.86, Fig. 3e). Conditional KM plots further revealed a longer OS for the CXCL13-high group compared to the CXCL13-low group in ARID1A-mut (*p* value = 0.012, adjusted *p* value (Bonferroni) = 0.048, log-rank test) (Fig. 3g, upper 2 panels). Meanwhile, the ARID1A-mut group showed a trend of longer OS than the ARID1A-wildtype group in both CXCL13-high and CXCL13-low subgroups (Fig. 3g, lower 2 panels). No significant statistical interaction was observed between the two markers (Fig. 3f, p value = 0.4, Wald test of interaction term in the dual marker Cox model). Besides the descriptive statistic, visualization and comparison between subgroups of dual biomarkers, Cox model comparison showed that CXCL13/ARID1A dual marker model had significantly better fitness to the survival data than ARID1A single marker model (*p* value = 0.00047, likelihood ratio test) and CXCL13 single marker model (*p* value = 0.1, likelihood ratio test). The combination of these two biomarkers achieved a higher power of risk discrimination as suggested by the bigger CPE value of the dual marker (CPE is 0.583, 0.535 and 0.576 for dual-marker, ARID1A and CXCL13 single marker, respectively). Therefore, the combination of these two biomarkers might suggest improved patient characterization and stratification. All plots, statistic result of LRT, CPEs were generated by running single function *dm_pair.*

### De novo identification of marker pairs

In *dualmarker*, given one specific marker (M1), another biomarker (M2) can be de novo identified using logistic regression for response status analysis and Cox models for survival analysis. An ideal dual marker model should be superior to either M1 alone or M2 alone. Therefore, we compared dual-marker models with M1 and M2 single marker models. Dual-marker models with and without interaction terms were both considered, and the model with smaller *p* value based on the LRT test was taken. The significance level (-log10 *p* value) for the dual-vs-M1 and dual-vs-M2 model comparisons is shown in the scatterplot in Fig. 4a. Goswami S et al. reported the combination of ARID1A mutation and CXCL13 expression predicts OS in three independent cohorts based on biological hypotheses [6]. We searched among all gene expression biomarkers for combination with the ARID1A mutation (mut_ARID1A) in a data-driven manner. Using the *dm_searchM2_cox* function, we identified 97 genes (*p* value < 0.01, LRT test) as dual-biomarker partner candidates to combined with mut_ARID1A, including CXCL13 and other important immune modulators such as IFNG and CXCL9/10 (Fig. 4b). These genes represent the inflamed immune signature that contributes to the association between ARID1A and overall survival. We also found some markers, such as HMGB1, when combined with the ARID1A mutation, outperformed either ARID1A or HMGB1 single marker model (*p* value = 0.0012, *p* value = 0.0003, likelihood ratio test, Fig. 4a); Meanwhile, HMGB1 expression showed an interesting statistical interaction with the ARID1A mutation. Under HMGB1-high conditions, patients with ARID1A mutation had significantly longer OS (Quadrant R1 vs R2, *p* value = 0.0015, log-rank test); However, this association disappeared in the HMGB1-low population (Quadrant R3 vs R4, Fig. 4d, p value = 0.48, log-rank test). The interaction term in the Cox model was also significant (*p* value = 0.001, Wald test), indicating that HMGB1 may have an important role in predicting OS with the ARID1A mutation. The relationship between HMGB1 and ARID1A needs further validation and exploration. We showed the nominal *p* values here, and no dual marker pairs were significant (FDR < 0.05) after *p* value adjustment using “BH” method, which may owe to the data set or the power of detection. However, such *p* value based prioritization strategy might identify interesting biomarker pairs before intensive study and validation.

## Discussion

Biomarker combination may increase the accuracy of efficacy prediction for cancer therapy via precise characterization and further stratification of patients. Such analysis might also reveal novel insight into the complicated cancer-TME interaction and help inform treatment strategy. Thus, an exploratory analysis tool for dual marker combination is valuable for streamlining the workflows.

To our knowledge, *dualmarker* is the first comprehensive R package for dual marker analysis. *dualmarker* involves various flexible and easy-to-use functions for dual marker characterization with two modules, generating over 14 types of plots to evaluate and visualize one biomarker pair. Here, we demonstrated its application in the well-studied Imvigor210 dataset and benchmarked the results with the original trial paper for the biomarker-pair example of the TMB/TGF-beta signature and ARID1A-mut/CXCL13 expression. Furthermore, powerful dual-marker pair searching functions can prioritize novel biomarker partners to use in combination with a pre-selected marker (M1). We searched key TME-related GEP markers in combination with ARID1A mutations using the Cox survival model and verified CXCL13 and IFNG as top candidates. We have also identified a novel statistically significant interaction between HMGB1 expression and the ARID1A mutation, representing that ARID1A mutated patients were with longer OS only in the HMGB1-high subgroup, while not in the HMGB1-low subgroup (Fig. 4). This interaction would require further validation study.

*Dualmarker* is a powerful exploratory tool for biomarker data mining. It streamlines potential dual-marker visualization and identification; Its strengths are as follows: (1) comprehensive visualization of marker pairs with over 14 types of plots, including boxplots, scatterplots, ROCs, and Kaplan–Meier plots; (2) comprehensive performance evaluation of dual marker combination. It builds four regression models and summarizes the model parameters simultaneously; Meanwhile it provides comprehensive model performance evaluation, including ROC/AUC for binary response variable, concordance probability for time-to-event variable, model comparison of dual marker versus single marker in logistic regression and Cox regression. (3) novel biomarker pair identification function without predefined hypotheses; (4) applicable to both survival and response analyses and compatible with both continuous and categorical variables; (5) user friendly with only 5 core functions. The interface for dual marker visualization and statistics of response and survival is *dm_pair,* which is compatible with both continuous (e.g., gene expression, TMB) and categorical (e.g., mutation, PDL1 level) variables. De novo marker2 identification is achieved through *dm_searchM2_logit* and *dm_serach_M2_cox* for logistic and Cox regression, respectively; all dual biomarker combination can be evaluated using *dm_combM_logit* and *dm_combM_cox* function. A detailed and up-to-date tutorial can be found at https://github.com/maxiaopeng/dualmarker. Thus, we expect wide usage of *dualmarker* in biomarker studies for the evaluation of biomarker pairs of interest and exploration of novel biomarker combinations for precise patient stratification as well as mechanism of action and resistance research.

*Dualmarker* is designed for data visualization and exploration purpose with some limits. Firstly, it can’t deal with the non-linear associations between predictors and the outcome for the four regression models, which needs methods such as restricted cubic spline and may be included in the package of the future version. Secondly, the regression model can include covariates, however, the current version can’t deal with the interactions between covariates and biomarkers. Thirdly, the package can’t handle ordinal variables, which need dichotomization aforehand, and it is not feasible for multiple (> 2) marker analysis. Finally, evaluating all marker combination from thousands of biomarkers like gene expression profiles, is time-consuming, and parallel computation or other methods could be introduced to improve the computation efficiency.

## Conclusions

A new open-source software toolkit, which includes an R package *dualmarker* and accompanied tutorial, is presented. This package implements logistic regression and Cox survival analysis models, and the data visualization command can be easily applied for biomarker pair evaluation and novel biomarker combination identification.

### Availability and requirements

- Project name: *dualmarker*
- Project home page: https://github.com/maxiaopeng/dualmarker
- Operating system(s): Platform independent
- Programming language: R
- Other requirements: ggplot2, dplyr, pROC, survival, survminer, CPE, ggpubr
- License: MIT License
- Any restrictions to use by non-academics: no restrictions

## Data Availability

*Dualmarker* is freely available under the MIT license through the link https://github.com/ maxiaopeng/dualmarker. The data that supports the findings of this study is available from IMvigor210 study (http://research-pub.gene.com/IMvigor210CoreBiologies/) with Creative Commons-BY-3.0 license.

## Abbreviations

AUC: Area under the curve
CPE: Concordant probability estimate
GEP: Gene expression profiling
ICB: Immune checkpoint blockade
OS: Overall survival
ROC: Receiver operating characteristic
TMB: Tumor mutational burden
